# Effects of MDMA Injections on the Behavior of Socially-Housed Long-Tailed Macaques (*Macaca fascicularis*)

**DOI:** 10.1371/journal.pone.0147136

**Published:** 2016-02-03

**Authors:** Sébastien Ballesta, Gilles Reymond, Matthieu Pozzobon, Jean-René Duhamel

**Affiliations:** 1 Centre de Neuroscience Cognitive, Centre National de la Recherche Scientifique, 69675 Bron, France; 2 Département de Biologie Humaine, Université Lyon 1, 69622 Villeurbanne, France; University of Chicago, UNITED STATES

## Abstract

3,4-methylenedioxy-N-methyl amphetamine (MDMA) is one of the few known molecules to increase human and rodent prosocial behaviors. However, this effect has never been assessed on the social behavior of non-human primates. In our study, we subcutaneously injected three different doses of MDMA (1.0, 1.5 or 2.0mg/kg) to a group of three, socially housed, young male long-tailed macaques. More than 200 hours of behavioral data were recorded, during 68 behavioral sessions, by an automatic color-based video device that tracked the 3D positions of each animal and of a toy. This data was then categorized into 5 exclusive behaviors (resting, locomotion, foraging, social contact and object play). In addition, received and given social grooming was manually scored. Results show several significant dose-dependent behavioral effects. At 1.5mg/kg only, MDMA induces a significant increase in social grooming behavior, thus confirming the prosocial effect of MDMA in macaques. Additionally, at 1.5 and 2.0 mg/kg MDMA injection substantially decreases foraging behavior, which is consistent with the known anorexigenic effect of this compound. Furthermore, at 2.0 mg/kg MDMA injection induces an increase in locomotor behavior, which is also in accordance with its known stimulant property. Interestingly, MDMA injected at 1.0mg/kg increases the rate of object play, which might be interpreted as a decrease of the inhibition to manipulate a unique object in presence of others, or, as an increase of the intrinsic motivation to manipulate this object. Together, our results support the effectiveness of MDMA to study the complex neurobiology of primates’ social behaviors.

## Introduction

A broad landscape of biological and cognitive mechanisms is dedicated to the management of animals’ social behaviors. In non-human primates’ affiliative behaviors are believed to be expressed in particular by social grooming behavior [1–4]. However the full range of biological factors modulating the occurrence of such genuinely social interactions is still not fully characterized [5]. Indeed, few pharmacological modulations has been shown to significantly increase the occurrence of affiliative behaviors in non-human primates [6–9]. In Humans, some chemicals induce a “specific altered state of consciousness with emotional and sensual overtones” [10], this effect is called entactogenic [11] and is produced principally by molecules composed of phenethylamine core. In particular, 3,4-methylenedioxy-N-methyl amphetamine (MDMA, aka “ecstasy”) has been reported to induce, *inter alia*, a salient increase in empathy, understanding and feelings of other closeness [12,13]. The neurobiology of entactogens mainly involves a modulation of the metabolism of monoamine signaling [14]. MDMA specifically stimulates serotonin, dopamine and noradrenalin efflux by altering the functioning of their respective transporters [15–18]. Besides that, MDMA also displays a micromolar affinity to some noradrenergic, serotoninergic, muscarinic, histaminergic and dopaminergic receptors [19,20]. The complex neurobiology of MDMA, which could be partially explained by a differential effect of each of its enantiomer [21,22], also involves second order secretion of hormones such as prolactin, oxytocin and cortisol [23,24]. It has been suggested that that some of the subjective effects of MDMA are specifically driven by these hormonal modulations [13,25–29]. In rodents, for instance, an increase of the amount of adjacent lying behavior following an MDMA administration was correlated with an activation of the oxytocinergic neurons, likely through serotoninergic signaling and especially 5-HT1a receptors [30–32].

When considering projection pathways and receptor localization, the serotoninergic system of macaques is broadly similar to that of humans [33]. Non-human primates might thus be useful to bridge the neurobiology of entactogens in rodents and in humans. Many studies using single-housed non-human primates have focused on the deleterious potential of MDMA on serotoninergic projections [34–38]. However, the effect of MDMA administration on the social behavior of non-human primates has never been assessed quantitatively. In this experiment, we used a custom-designed multi-camera 3D tracking system [39], to record for extended periods of time the behaviors of 3 socially housed males juvenile long-tailed macaques (*Macaca fascicularis*) following a subcutaneous injection of either a saline solution or of MDMA at three doses (1.0, 1.5 or 2.0 mg/kg).

## Materials and Methods

### Animals

Three non-kin but group-housed juvenile male long-tailed macaques (aged 3+/-0.15 years, weight 5.7+/-0.8) were used as subjects. They were housed as a mini-colony in a large enclosure (15m^3^) allowing direct physical interaction, but also to isolate the monkeys when needed by means of a system of sliding partitions. When isolated, the monkeys could communicate visually and vocally at all times. Animals were fed with monkey chow, fresh fruits and vegetables. The cages were enriched with ropes, mirrors and woodchips to promote foraging. During behavioral recordings, the presence of objects in their cage was carefully controlled, so that the objects inserted at the beginning of each recording session was the only one of interest present in the cage. Animals’ behavior following drug treatment was monitored continuously thanks to our in house 24/7 video-surveillance system. This study was approved by the local animal experimentation ethics committee (CELYNE) and used experimental procedures complying with the recommendations of the local authorities on Animal Care (Direction Départementale des Services Vétérinaires, Lyon, France) and the European Community standards for the care and use of laboratory animals [European Community Council Directive (1986), Ministère de l’Agriculture et de la Forêt, Commission Nationale de l’Expérimentation Animale]. This study was also supervised by the Cognitive Neuroscience Center’s Animal Welfare Committee. At the present time, all tested animals are still alive and used for behavioral observation studies.

### Chemicals

Chemicals were sourced from Sigma-Aldrich with governmental authorizations from the ANSM (Agence nationale de sécurité du medicament et des produits de santé, Autorisation n°: A-2013-6-738-S) and properly stored in a restricted area.

### Behavioral procedure

In order to reduce the stress of the injection, the animals were extensively trained prior to the experimental test with fake injections and positive reinforcement using clicker training. The experimental procedure started with an animal being isolated and injected subcutaneously with either 500μl of saline or MDMA at three possible doses (1.0, 1.5 and 2.0 mg/kg). These doses were based on those known to produce a subjective and physiological effect in Humans [23] and monkeys [40] while respecting posology known not to produce any measurable damage to the serotoninergic projections of the animals [34]. Indeed, for each animal, an interval of at least one week was respected between successive MDMA injections. During experimental sessions, one animal was injected with MDMA while the two others were injected with saline [41]. After the injection, a unique colored toy was introduced inside the home cage and left until the next morning. The recording sessions started at 5 p.m. and ended 3 hours later with the gradual extinction of the light. These procedures were performed over a 3-month period.

### Automatic and manual behavior measures

We used a custom-designed multi-camera 3D tracking system [39], to record and monitor the behavior of primates in their living space. This system can track the location of multiple animals in real-time, provided they are wearing a unique color marker (restraining collar or head-post). Animal positions (X, Y, Z) were estimated by triangulation from the set of image coordinates of their respective color targets when viewed by at least 2 cameras. Measurements for 3 animals and 1 colored toy were taken simultaneously at 15 Hz rate, with a nominal spatial accuracy of 1 cm. Position recordings were then processed to derive animal relevant behavioral measurements, except grooming which has been scored manually from raw videos. The sum of the time spent engaged in the seven recorded behaviors matches the whole duration of the recording sessions.

### Data analysis

The behavior of each animal injected with MDMA was compared with the same animal behavior during the closest control session (up to 4 days before the experimental session) thus allowing the use of pair-wise non parametric statistics (Wilcoxon signed-rank test). Data analysis and statistics were performed using custom scripts written in Matlab R2010.

## Results

**Fig 1:**
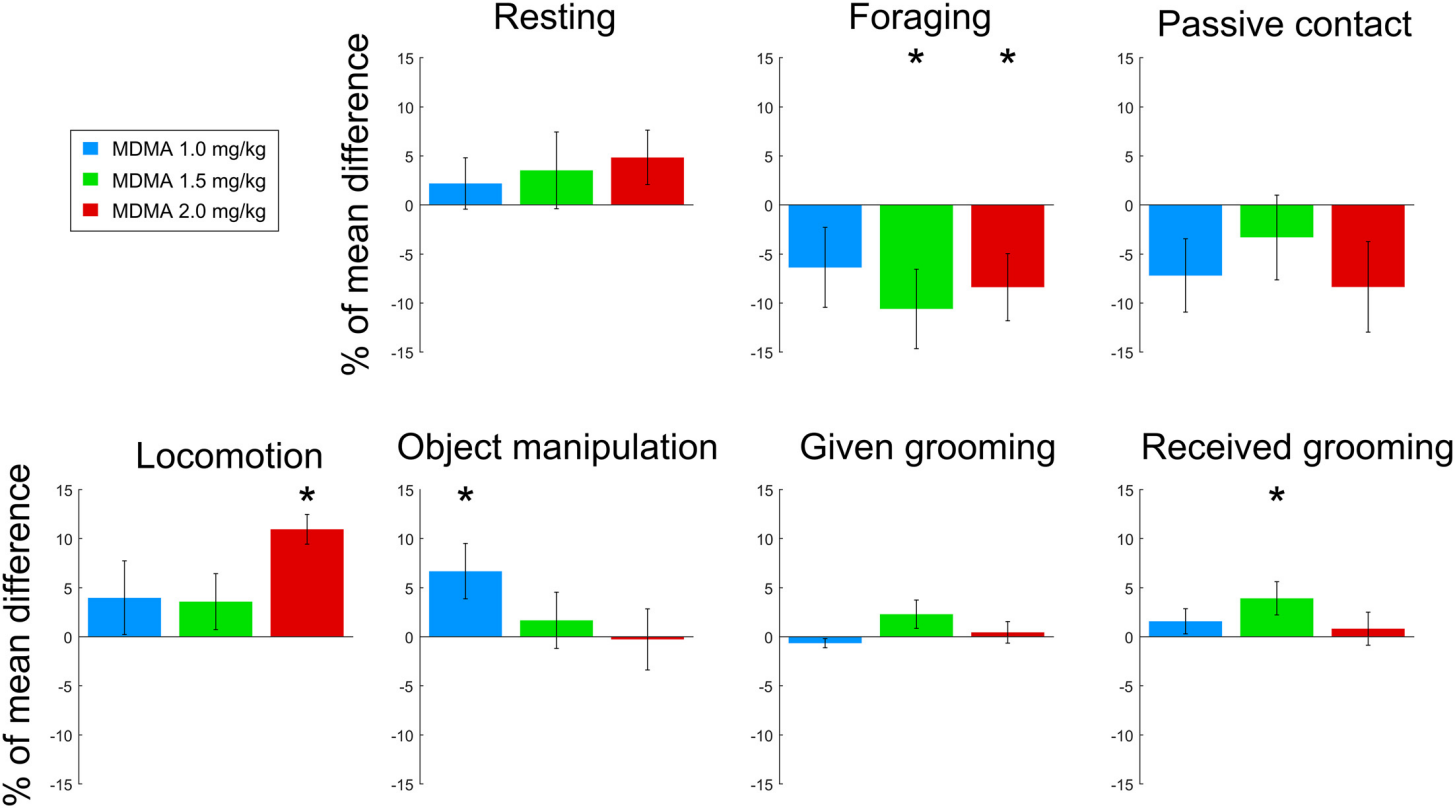
Effects of MDMA on spontaneous behavior. Mean difference in frequency of each measured behavior between MDMA injections and their respective saline control injections. Positive values mean that the behavior was increased by MDMA injection. Error bars represent the SEM. 10% of time is equal to 18minutes. Number of sessions: MDMA 1.0 mg/kg = 11, MDMA 1.5 mg/kg = 14, MDMA 2.0 mg/kg = 9. * indicates significant differences (Wilcoxon signed-rank test, p<0.05).

**Fig 2:**
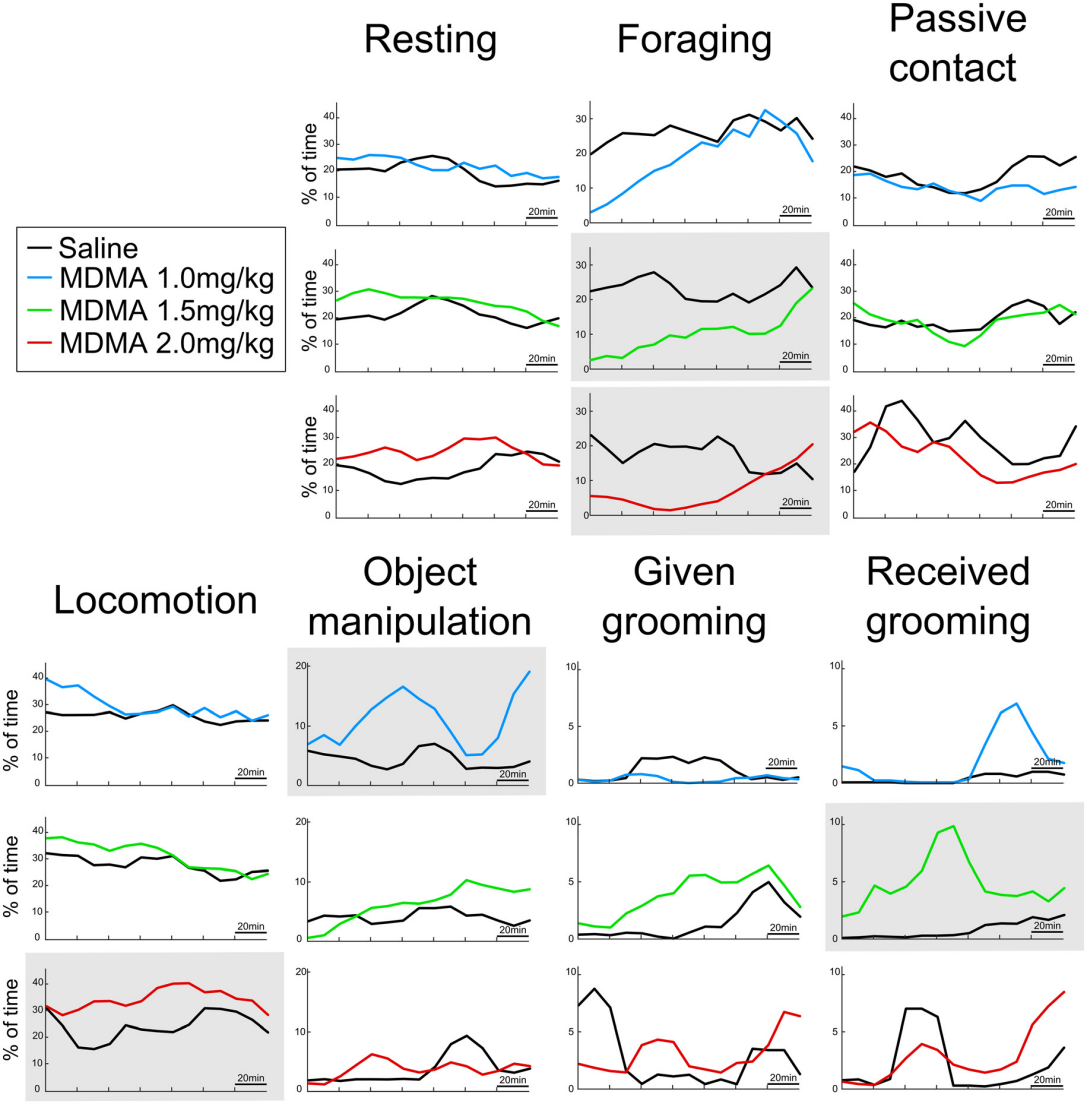
Time course of all behaviors after MDMA injection. Number of sessions: MDMA 1.0 mg/kg = 11, MDMA 1.5 mg/kg = 14, MDMA 2.0 mg/kg = 9. Grey overlay indicate an overhaul significant differences between the two conditions (Wilcoxon signed-rank test, p<0.05).

Our methodology allowed to score the amount of time allotted to 7 distinct behaviors: locomotion, resting, foraging (animal on the ground looking for goods), object manipulation (interdistance with the object < 20cm), grooming given, grooming received and social contact (interdistance < 30cm with a peer, grooming excluded). The mean activity budget of the considered animals for all 3-hours saline control sessions is presented in S1 Fig. The differences between animals’ behaviors when injected with MDMA versus saline are presented in Fig 1. Additionally, individual data are presented in S2 Fig. We did not quantify abnormal behaviors such as stereotypy but the treatments seemed to be well tolerated by the animals as no adverse events were detected. Several significant dose-dependent behavioral effects have been found. At small dose (1.0 mg/kg), MDMA injection only increased significantly object manipulation (Wilcoxon signed-rank test, p<0.05). At medium dose (1.5mg/kg), MDMA injection significantly decreased foraging behavior while increasing the time spent being groomed by a conspecific (Wilcoxon signed-rank test, p<0.05). At large dose (2.0 mg/kg), MDMA injection significantly decreased foraging behavior while producing a large increase in locomotor behavior (Wilcoxon signed-rank test, p<0.05). In addition, together with other non-significant conditions, the time course of these significant effects is presented in Fig 2. The effect of the medium dose of MDMA on grooming behavior reached its maximum around 100 minutes after the injection. The effect of the small doses of MDMA on object manipulation behavior contains two distinct peaks, one around 80 minutes post injection, and the other at the end of the recording sessions, while the control session only displays one peak at around 100 minutes post injection. At both medium and large doses, MDMA injections induced a rapid and long lasting inhibition of foraging behavior. At large doses, MDMA induced an increase in time spent in locomotor activity reaching its maximum at around 120 minutes post-injection.

## Discussion

Despite our relatively small number of subjects, the measured dose-dependent behavioral effects of MDMA injection on juvenile male long-tailed macaques are mostly consistent with its known effects (e.g. increase of social affiliation, activity, and a decrease of hunger) on rodent and human behavior. As a member of the methamphetamine family, a large dose of MDMA injection has a stimulant effect via releasing of norepinephrine and stimulation of adrenergic alpha 1 receptors [42–44]. Interestingly, previous studies report an opposite effect of MDMA on the locomotor activity of rhesus macaques [35,45,46]. Such contradiction might be explained by the fact that in previous studies, macaques were housed individually. In our study, the mere presence of others might have favored the known stimulant effect of MDMA. Methamphetamines are also known to induce an anorexigenic effect, which is fairly consistent with the long-lasting observed decrease in foraging after the injection of medium and large doses of MDMA. It is worth noticing that the use of foraging behavior might represent a suitable model of consummatory (and maybe exploratory) behaviors in macaques. The effect of small doses of MDMA upon object play behavior could be interpreted in different ways, however the lack of data available on the hormonal correlates of solitary object play and the physiological consequences of small dose of MDMA injection makes it hard to distinguish among these. A possible explanation for the observed increase in object play at low MDMA doses could be a decrease of social fear, based on previous findings showing the influence of peers on object play [47–49]. However, the fact that small dose of MDMA has little impact on social contact or grooming behaviors is not in favor of this explanation. Another interpretation could be that at small dose, MDMA modulates the intrinsic motivation to manipulate objects. This might be driven by a genuine increase of curiosity, a need to alleviate a stress, or a hallucinogen-like effect. However, none of these explanations are likely based on the other observed behaviors. In our experiment, a small dose of MDMA did not significantly increase foraging behavior, which is inconsistent with a general increase in curiosity. Even if it has been shown in previous a study of [50], a possible increase in anxiety by MDMA injections seems also unlikely to explain a modulation of object play behavior. Finally, MDMA produces hallucinogen-like effects only at high dosage [51,52], and since in our study, medium and large doses did not affect object play, it is also unlikely that this increase of object play has been triggered by such an hallucinogen-like effect Therefore, further investigation seems necessary to reliably explain this interesting increase of primates’ object-oriented behavior. Interestingly, at medium dosage, MDMA induced an increase of social grooming, an effect of undeniable prosocial nature. This result is consistent with the known effect of MDMA on rodent and human social behaviors, and thus highlights an evolutionary continuity in mammals’ hormonal control of social behavior. However, MDMA only increases received but not given social grooming, which suggest that such prosocial effect might be explained by an increase of non-aggressive postures more than by a genuine increase in motivation for social affiliation [53]. A recent study has shown that sertraline (a selective serotonin reuptake inhibitor) administration on macaques also induce a dose-dependent increase of grooming behaviors [8], hence the observed prosocial effects of MDMA could be mediated by a modulation of the serotoninergic system. The fact that that blocking MDMA-induced release of serotonin markedly reduces the entactogenic response to MDMA in humans also supports the implication of the serotonin system in producing such prosocial response [29,54]. However, the characteristics of entactogens are unlikely to be explained by their effects on a single neuromodulator. Indeed, as the outcome valence of social interactions is not always predictable, the control of social behavior needs to involve dynamic internal and external variables that probably require complex neuronal and hormonal regulators. A synergistic action of different hormones on the social brain might be one of such mechanisms. Amongst the numerous hormones implicated in the regulation of social behavior, prolactin, oxytocin and cortisol has been suggested to be responsible for entactogenic modulation [8,13,27]. Prolactin is implicated in social rewards process [55], oxytocin is involved in the modulation of social motivation and attachment [56–58] and cortisol is considered as an energizing hormone which might help to deal with the metabolic needs related to the intrinsic unpredictability of social interactions [24]. Hence, we believe that the involvement of these three hormones in the neurobiology of entactogens make them good candidate to be also implicated in the physiological regulation of mammals’ social behaviors. However, further empirical proofs are necessary to demonstrate that a causal link exists between this hormonal cocktail and specific entactogenic effect.

To sum up, our study shows that MDMA injected at 1.5mg/kg would be a suitable procedure to refine our understanding of the complex neurobiology of primates’ social behaviors. We further suggest that solitary object play might be of interest when studying primate behavior.

## Supporting Information

S1 ARRIVE Checklist NC3R Animal Research: Reporting In Vivo Experiment Guidelines Checklist. (PDF)

S1 Fig Means of behaviors measured automatically and manually during 3-hour recording sessions following a saline injection (n = 34 sessions). Error bars represent the SEM. 10% of time is equal to 18 minutes.
(TIF)

S2 Fig Effects of MDMA on spontaneous behavior of each individual. Positive values mean that the behavior was increased by MDMA injection. * indicates significant group differences (Wilcoxon signed-rank test, p<0.05). Error bars represent the SEM.
(TIF)
