# Supplementary figures and images for: Effects of MDMA Injections on the Behavior of Socially-Housed Long-Tailed Macaques (Macaca fascicularis)
Source: PLoS One. 2016 Feb 3;11(2):e0147136. doi: 10.1371/journal.pone.0147136 (PMC4739726; doi:10.1371/journal.pone.0147136)

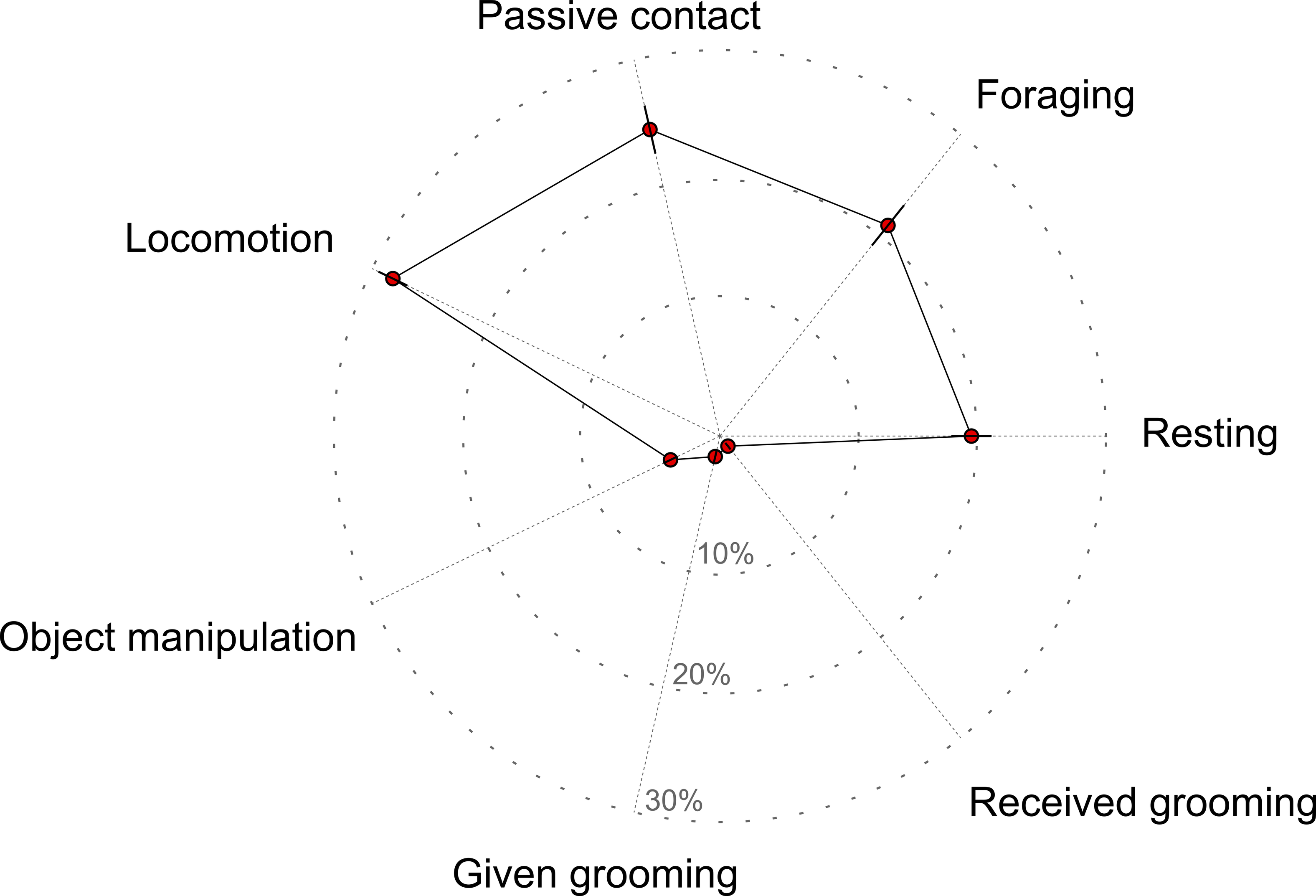

Supplement: S1 Fig — Error bars represent the SEM. 10% of time is equal to 18 minutes. (TIF) [file pone.0147136.s002.tif]

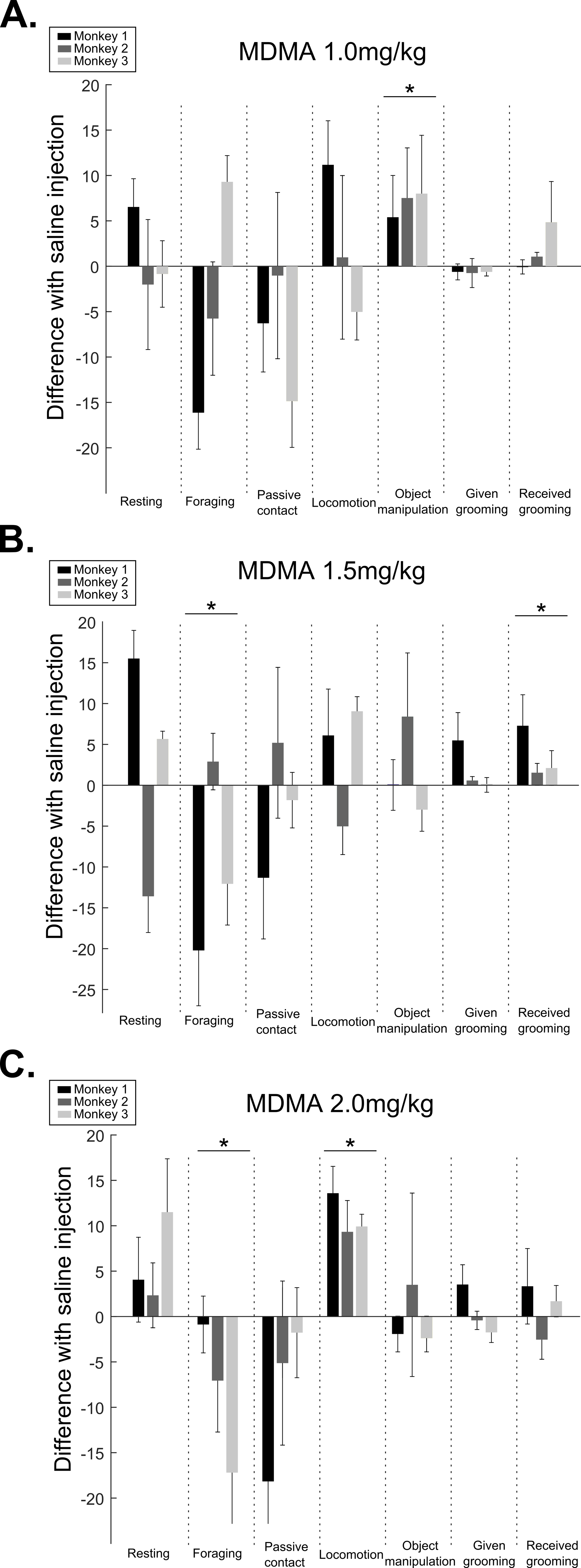

Supplement: S2 Fig — Positive values mean that the behavior was increased by MDMA injection. * indicates significant group differences (Wilcoxon signed-rank test, p<0.05). Error bars represent the SEM. (TIF) [file pone.0147136.s003.tif]
